# Deciphering Heterogeneity in Pig Genome Assembly Sscrofa9 by Isochore and Isochore-Like Region Analyses

**DOI:** 10.1371/journal.pone.0013303

**Published:** 2010-10-11

**Authors:** Wenqian Zhang, Wenwu Wu, Wenchao Lin, Pengfang Zhou, Li Dai, Yang Zhang, Jingfei Huang, Deli Zhang

**Affiliations:** 1 Bioinformatics Center, College of Life Science, Northwest A&F University, Xianyang, Shaanxi, China; 2 Investigation Group of Molecular Virology, Immunology, Oncology and Systems Biology, and Bioinformatics Center, College of Veterinary Medicine, Northwest A&F University, Xianyang, Shaanxi, China; 3 State Key Laboratory of Genetic Resources and Evolution, Kunming Institute of Zoology, Chinese Academy of Sciences, Kunming, Yunnan, China; Deutsches Krebsforschungszentrum, Germany

## Abstract

**Background:**

The isochore, a large DNA sequence with relatively small GC variance, is one of the most important structures in eukaryotic genomes. Although the isochore has been widely studied in humans and other species, little is known about its distribution in pigs.

**Principal Findings:**

In this paper, we construct a map of long homogeneous genome regions (LHGRs), i.e., isochores and isochore-like regions, in pigs to provide an intuitive version of GC heterogeneity in each chromosome. The LHGR pattern study not only quantifies heterogeneities, but also reveals some primary characteristics of the chromatin organization, including the followings: (1) the majority of LHGRs belong to GC-poor families and are in long length; (2) a high gene density tends to occur with the appearance of GC-rich LHGRs; and (3) the density of LINE repeats decreases with an increase in the GC content of LHGRs. Furthermore, a portion of LHGRs with particular GC ranges (50%–51% and 54%–55%) tend to have abnormally high gene densities, suggesting that biased gene conversion (BGC), as well as time- and energy-saving principles, could be of importance to the formation of genome organization.

**Conclusion:**

This study significantly improves our knowledge of chromatin organization in the pig genome. Correlations between the different biological features (e.g., gene density and repeat density) and GC content of LHGRs provide a unique glimpse of *in silico* gene and repeats prediction.

## Introduction

A number of studies [1]–[4] have revealed that eukaryotic genomes of warm- and cold-blooded vertebrates, and even a few plants, are mosaics of isochores. The term isochore refers to a relatively long DNA segment (above 300 kb on average) that has a fairly homogeneous (either GC-rich or AT-rich) base composition (above 3 kb in size), as well as sharp boundaries with neighboring isochores [2], [5]. According to different levels of GC content, isochores can be assigned to a number of families. Although the origin of isochores has not yet been fully clarified, some evidence indicates that the isochore structure is closely connected with chromosome bands, as well as many important biological properties including gene density, repeat sequence distribution, CpG distribution, and replication timing [2]. Hence, the isochore pattern greatly increases our appreciation of the compositional heterogeneity and the complexity of eukaryotic genomes [6] and is now widely recognized as “a fundamental level of genomic organization” [7].

Two of the foremost problems in isochore research are the identification of isochore boundaries and the definition of homogeneity; hence, a variety of isochore assignments have been proposed to resolve the two issues [8]–[14]. However, assignments of the sa`me sequence occasionally differ among the different methods [15], since the criteria for isochore homogeneity vary widely among these methods. As a result, some isochore-like regions, which have somewhat less-constant but significantly more-heterogeneous GC contents relative to the adjacent regions, may be neglected by some methods. To better understand the compositional features of the genome, the method of non-overlapping long homogeneous genome regions (LHGRs) [16] is proposed to reflect homogeneities and heterogeneities, not only in the isochores, but also in the isochore-like regions in each chromosome.

The pig (*Sus scrofa*) is an economically important species and is an excellent medical model for humans due to the extensive similarities between the two species. Early studies [17], [18] that employ compositional DNA fractionation and *in situ* hybridization have shown that the pig genome is compositionally similar to the human genome. The pig genome also has isochores belonging to the five known families [2], [10], however, further details about the isochore pattern, such as numbers and boundaries at base resolution, have not yet been determined. Luckily, the availability of a high coverage (4×) assembly of the pig genome released in September 2009 now provides an unprecedented chance to explore novel compositional features in the pig genome.

The goals of this study are: (1) to evaluate the LHGR architecture and pattern in the pig genome, (2) to compare the compositional heterogeneities between the pig and human genomes, and (3) to identify the relationship between LHGRs and gene/repeat density. Here, we initially determine the locations of 2,491 LHGRs in the pig genome, as well as 2,568 LHGRs in the human genome. All pig LHGRs are then classified into isochores and isochore-like regions. Thereafter, we describe the architecture of the LHGRs in each chromosome by z' curves [19] to simultaneously reveal the gradual and abrupt LHGR boundaries. By examining the LHGR patterns, including the proportions and size distributions of the five LHGR families, we find some compositional features displaying the same patterns as in warm-blooded vertebrates. Relatively similar LHGR patterns between pigs and humans provide evidence of the compositional similarity between the two species. Moreover, we find the evidence of the correlation between LHGRs and some biological sequences, such as genes and LINEs, which have been observed experimentally in portions of pig chromatins [17].

## Results

### z' curves for 19 pig chromosomes

In comparison to traditionally sliding-window-based method [2], z' curve is a windowless tool used to illustrate intuitively the GC content fluctuations in a sequence. Deviation of any point from the z' curve is inversely proportional to the GC content of the corresponding site in a sequence [19].

**Figure 1:**
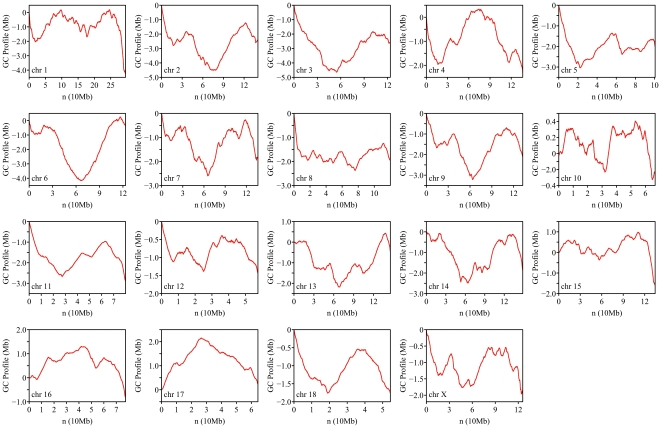
z' curves for 19 pig chromosomes. A positive slope indicates a decrease in GC content, whereas a negative slope indicates an increase in GC content. Each curve is composed of regions that either are approximately straight (referred to as isochores) or have large fluctuations (referred to as isochore-like regions).

The z' curves of pig chromosomes (Figure 1 and S1) indicated that the GC content along the chromosomes were heterogeneous, inasmuch as each curve underwent dramatic fluctuations. However, in these curves, there were some regions that approximately fit straight lines, indicating that these regions had nearly constant GC contents. Such regions could be regarded as isochores, whereas other regions that showed pronounced fluctuations could be regarded as isochore-like regions [20]. In fact, when the curves were divided into sufficiently small segments, they could be considered as approximately straight lines; the regions corresponding to the straight lines were then referred to as LHGRs (Figure S1). Therefore, non-overlapping LHGRs along each chromosome were comprised of isochores and isochore-like regions (see detailed classification of LHGRs in Materials and Methods).

According to the slopes of the straight lines on z' curves, all of the LHGRs could be divided into two types: AT-LHGRs and GC-LHGRs. As shown in Figure 1, a negative slope represents a higher GC content in one LHGR compared to the average GC content of the chromosome. Thus, LHGRs with negative slopes were designated as GC-LHGRs, whereas those with positive slopes were designated as AT-LHGRs [4].

### LHGR mapping

**Figure 2:**
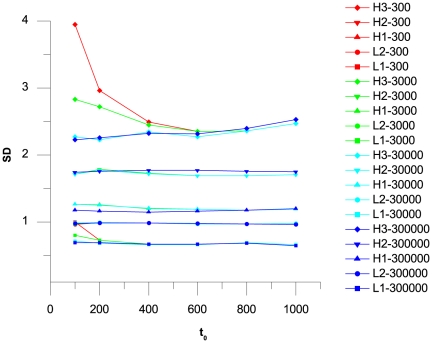
Plots of the SD value of the GC content within each LHGR family against 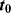 and 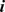. Plots are shown for all of pig LHGRs, produced under the given threshold and partitioned into five families according to GC contents. Colors and labels of these curves stand for different 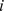 values and families, respectively. Among all the LHGRs, the H3 family has the greatest SD variation. When both 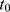 and 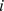 are set to smaller values in the H3 and L1 families, larger SD values are observed.

In the present study, GC-Profile [11] was applied to divide the genome into LHGRs using the segmental halting parameter (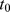) and the minimum length (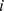), which were equal to 100 and 300,000, respectively (see Materials and Methods). The two parameters were chosen because the plots of the average standard deviations (SD) of the GC content against 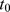 and 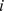 (Figure 2) indicated that their SD values increased following an increase in the GC content of the family, but dropped when the 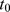 and 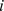 values were 100 and 300,000, respectively.

As a result, a total of 2,491 LHGRs were identified in the pig genome (Table 1), as well as 2,568 LHGRs in the human genome. Furthermore, 1,204 LHGRs, nearly half of the pig LHGRs, were classified as GC-LHGRs, and the rest were classified as AT-LHGRs (Table S1).

**Table 1:** The length, GC content and number of LHGRs in 19 pig chromosomes.

chr No.	chr.Length (bp)	chr.Length (bp) (excluding gaps)	G+C content (%)	No. of LHGRs
1	295,534,705	291,978,373	40.32	321
2	140,138,492	138,150,962	42.16	162
3	123,604,780	121,517,467	43.86	142
4	136,259,946	135,000,183	41.44	168
5	100,521,970	98,837,867	41.65	116
6	123,310,171	121,214,429	44.41	150
7	136,414,062	135,056,527	43.17	157
8	119,990,671	117,775,145	39.42	117
9	132,473,591	130,657,580	41.05	134
10	66,741,929	65,778,119	42.25	70
11	79,819,395	78,559,732	40.43	79
12	57,436,344	56,502,398	46.96	71
13	145,240,301	142,767,306	39.96	161
14	148,515,138	147,418,116	43.24	178
15	134,546,103	132,149,378	39.78	136
16	77,440,658	76,457,938	40.47	81
17	64,400,339	63,427,956	44.27	71
18	54,314,914	53,590,780	42.61	53
X	125,876,292	124,474,993	40.13	124

The distribution of compositional differences (ΔGC) between adjacent LHGRs in the pig genome was tested and an obvious skewed distribution was found in each family. As shown in Figure S2, the ΔGC value was asymmetrical, with dispersion skewed to the lower side of the median. The average ΔGC of the LHGRs was 4.24% in the pig genome and 3.83% in the human genome.

### Isochore mapping

The homogeneity of the GC content in each LHGR was evaluated by an index 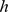 [4], defined by the division between GC content variances of the LHGR and the host chromosome where the LHGR was located. As a result, 342 LHGRs were classified into isochores, while 2,149 were classified into isochore-like regions (Table S1). The 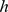 values of the isochores varied from 0.0015 to 0.1989; in contrast, the corresponding values of isochore-like regions ranged from 0.2022 to 3.5842. Of the 342 isochores, 80 were greater than 1 Mb in length, and the longest was 6.18 Mb. In addition, 151 of the identified isochores belonged to GC-poor families, whereas 191 belonged to GC-rich families. Table 2 lists 24 isochores in chromosome 16. More information, including the 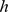 value, length, and classification of each LHGR, is listed in Table S1.

**Table 2:** Isochores in the chromosome 16 of pig.

No.	Start (bp)	Stop (bp)	Length (bp)	GC content (%)	Family	h
1	1	561,070	561,070	40.93	L2	0.0091
2	561,071	1,129,138	568,068	39.12	L2	0.0262
3	1,129,139	2,265,069	1,135,931	36.00	L1	0.0408
4	2,265,070	2,709,575	444,506	39.39	L2	0.0413
5	2,709,576	3,036,775	327,200	43.25	H1	0.0529
6	3,036,776	3,497,556	460,781	45.24	H1	0.0850
7	3,497,557	5,126,374	1,628,818	40.81	L2	0.1427
8	6,354,085	11,524,640	5,170,556	35.01	L1	0.1136
9	11,524,641	12,254,555	729,915	33.41	L1	0.0631
10	43,172,563	44,816,542	1,643,980	39.60	L2	0.0487
11	44,816,543	45,306,029	489,487	41.59	H1	0.0590
12	45,306,030	45,953,195	647,166	43.80	H1	0.0576
13	45,953,196	46,406,468	453,273	42.05	H1	0.0381
14	46,406,469	46,773,633	367,165	40.13	L2	0.0359
15	46,773,634	48,635,681	1,862,048	45.71	H1	0.0476
16	48,635,682	49,272,472	636,791	50.27	H2	0.0683
17	49,272,473	49,934,493	662,021	45.02	H1	0.0694
18	49,934,494	50,634,448	699,955	39.51	L2	0.0744
19	50,634,449	51,009,856	375,408	50.03	H2	0.1071
20	51,009,857	51,324,060	314,204	47.14	H2	0.1247
21	51,324,061	51,885,056	560,996	42.73	H1	0.1853
22	70,778,047	71,335,207	557,161	42.23	H1	0.1917
23	71,335,208	72,505,936	1,170,729	44.82	H1	0.0794
24	72,505,937	72,972,167	466,231	51.57	H2	0.1952

### LHGR pattern: the relative numbers

**Figure 3:**
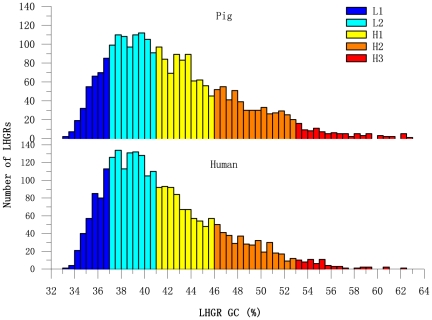
Number distributions of LHGRs according to GC content. The histograms show the distribution of LHGRs in bins of 0.5% GC content. The colors represent different LHGR families: L1 (blue), L2 (green), H1 (yellow), H2 (orange), and H3 (red).

When all the LHGRs in the pig and human genomes were pooled in bins of 0.5% GC content, the two species showed a high degree of similarity in the distribution of the five LHGR families; i.e., there was a regular decrease in the GC distribution of the LHGRs from GC-poor to GC-rich families. In Figure 3, the L2 and H1 families dominated the LHGRs, while the H3 LHGRs were scarce. In comparison to the human genome, the pig genome had a higher percentage of GC-rich LHGRs (see also Table 3a).

**Table 3:** The relative amount, average GC level and average size of LHGR families from pig and human (a, b, c).

	L1	L2	H1	H2	H3	Total
**(a) Relative amount (%)**						
**pig**	13.45	33.36	29.55	19.63	4.01	
**human**	15.69	38.58	27.96	15.22	2.56	
**(b) Average GC (%)**						
**pig**	35.80	38.98	43.23	48.94	56.24	42.48
**human**	35.87	38.94	43.18	48.74	55.27	41.55
**(c) Average size(Mb)**						
**pig**	1.31	0.89	0.86	0.79	0.68	0.91
**human**	1.57	1.08	1.09	0.92	0.95	1.20

### LHGR pattern: the size

**Figure 4:**
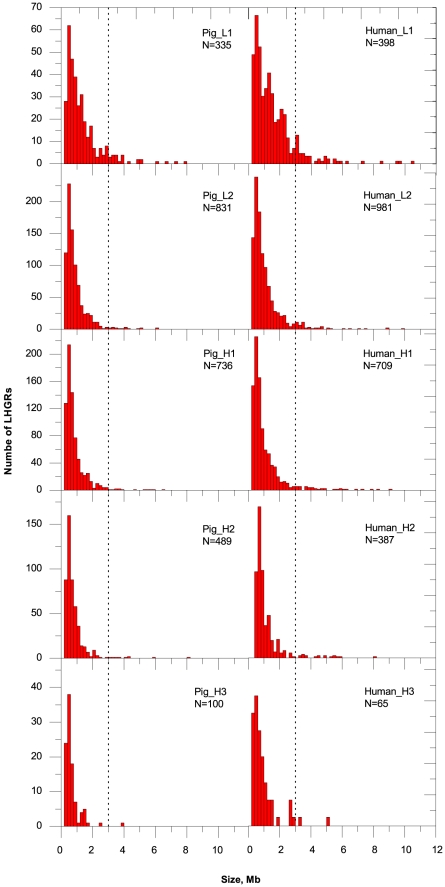
Size distributions of the LHGRs from their corresponding families. The histograms show the size distribution of each LHGR family in the pig and human genomes, and all of the LHGRs were pooled at intervals of 0.2 Mb. The vertical line at 3 Mb indicates the control.

LHGRs vary in size following the fluctuation of GC content. The strongly skewed size distributions of the LHGRs (Figure 4) in pigs and humans showed not only similarities but also differences between the corresponding LHGR families. The particular differences are the followings: (1) a smaller size (<1 Mb) and a narrower size distribution of the GC-rich LHGRs; and (2) a larger size (>3 Mb) and a wider size distribution of the GC-poorest LHGRs. The longest LHGR in pigs was localized in the chromosome 3 and was 8.08Mb in length (Table S1). Furthermore, the average size (0.91 Mb) of pig LHGRs was much shorter than that (1.20 Mb) of human LHGRs (Table 3b).

### Compositional distribution of pig genes

**Figure 5:**
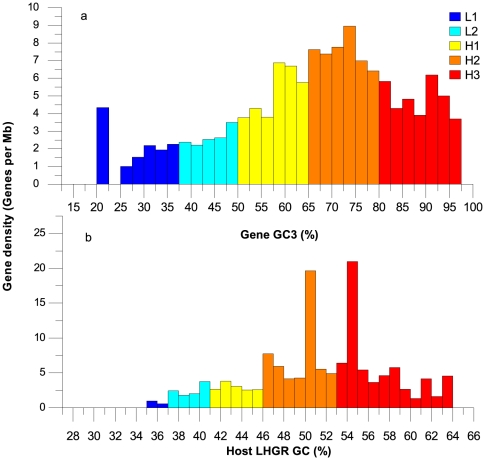
Compositional distribution of coding genes. a and b illustrate the gene density (the number of genes per Mb window) along the LHGRs according to gene GC3 and host LHGR GC, respectively. A total of 2,785 known coding genes from the pig genome were studied.

An association between gene density and GC content variation was recognized. In the study by Federico *et al.* [17], due to a lack of accurate isochore pinpointing, the gene densities in the pig isochores were examined indirectly using GC3 (the GC content at the third codon position). When the same GC3 criteria [17], i.e., L1 (GC3 %<37.5), L2 (37.5≤GC3 %<50), H1 (50≤GC3 %<65), H2 (65≤GC3 %<80), and H3 (GC3 %≥80), were applied to classify the LHGR families, the following result was observed: the pig gene density varied from very low in GC-poor families to very high in GC-rich families (Figure 5a). This conclusion was in accordance with the previous results reported for a considerable number of warm-blooded and cold-blooded vertebrate genomes [2], [17], [21]. However, the correlation (r^2^ = 0.35, p<10^−6^) between the gene GC3 and the host LHGR GC content showed that GC3 is somewhat an accurate index to assess the GC content of LHGRs (Figure S3), which is inconsistent with the report of Elhaik *et al.* [22]. To circumvent such possible problem, the compositional features of the pig genes were re-examined using the real GC contents of host LHGRs instead of the GC3. As shown in Figure 5b, the progression of gene density from GC-poor families to GC-rich families did not show the same smooth ascent as seen in Figure 5a. Furthermore, two t-test results showed that the gene densities in certain GC content ranges, H2 (50%–51%) and H3 (54%–55%), were significantly (both p<10^−6^) higher than in other ranges. Although the highest density still appeared in the H3 family, in accordance with the classification of all of the genes into the host LHGRs families, the number of genes residing in the H3 family was significantly (p<10^−6^) fewer than in other two GC-rich families (Figure S4).

### Density of repeats in LHGRs

**Figure 6:**
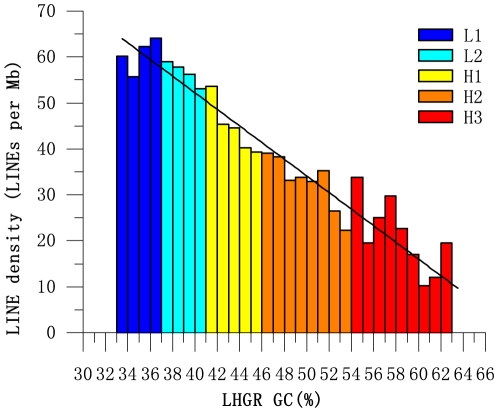
LINE density in LHGRs. A total of 120,870 LINEs were applied to the study of LINE density, which was calculated based on a 1 Mb non-overlapping sliding window. The straight line indicates the regular decrease in LINE density from GC-poor to GC-rich LHGRs.

The densities of Alu and LINE repeats vary with the changes in the GC content of isochores [23]. To investigate whether or not this relationship was also applicable to LHGRs, the variations in LINE density along LHGRs were analyzed in detail, whereas the Alu repeats were ignored because of the fewer number of data sets for the pig Alu repeats in Repbase [24]. As shown in Figure 6, the LINEs were frequent in L1 LHGRs, but practically absent in H3 LHGRs (r^2^ = 0.93, p<10^−6^), and the results followed the patterns previously found in isochores.

## Discussion

One challenge in the partition of complex eukaryotic genomes based on GC content is to find a set of parameters suitable for coping with the significantly different levels of GC fluctuations in the GC-rich and GC-poor regions. To reduce the fluctuations in GC content within each family, the SD value of the GC content in each LHGR family was first analyzed against the two important parameters (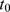 and 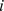 ) in the GC-Profile, after which the parameters that could produce the minimum SD value in each family were chosen. Thereafter, the GC difference between adjacent LHGRs was tested. The average ΔGC of the human LHGRs (3.83%) nearly reached the value (3.90%) obtained through the window method from Costantini *et al.* [10]. This result confirms the reasonability of the segmentation method on LHGRs used in the present study.

The number of LHGRs reflects the extent of homogeneity in a chromosome. In our study, the pig chromosome 12 is longer than chromosome 17, even though both chromosomes are divided into 71 segments (Table 1). This implies that chromosome 12 has a higher homogeneity than chromosome 17. Accordingly, the z' curve of chromosome 17 should fluctuate more substantially than that of chromosome 12. Indeed, this was confirmed by our z' curve assay (Figure 1).

The search for isochore patterns involves the assessment of two properties in each isochore family: the relative number and the average LHGR size. Analysis of these key LHGR characteristics reveals that the LHGR patterns in both the pig and human genomes follow the conservatively evolutionary isochore pattern, and display the general compositional pattern in mammalian genomes [17]. Both the distributions of relative number and average size of each LHGR family show a steady decrease from GC-poor families to GC-rich families. On average, a higher GC content in the pig genome (42.48%) was observed compared to the human genome (41.55%); however, the GC content of each LHGR family in the two species is relatively conserved (p<0.05). These conserved patterns may indicate some special functions relevant to chromatin structure [25]. Indeed, the number of LHGRs (2,568) estimated for the human genome is in agreement with the maximum number (3,000) assessed by Yunis *et al.* [26] using experimental methods of high resolution bands. The high proportion of GC-poor LHGRs is seemingly due to the preferred insertion of interspersed repeated sequences in these families, as well as the sequence expansion phenomena [26]. Moreover, the GC-skewed repeats also appear to explain the larger size and larger spread of the GC-poor LHGRs families (Figure 4). The presence of large gaps (more than 1% of the chromosome) in the human genome, but not in the pig genome, may also give rise to the long tail in the size distribution of human L1 LHGRs (Figure 4), which is virtually absent in the pig L1 LHGR distribution. This implies that more complete sequence data will be needed to obtain a reliable comparison of the size of the GC-poorest LHGRs between the pig and human genomes.

The conservation mode of isochore evolution was originally explained by “negative selection acting at a regional (isochore) level to eliminate any strong deviation from the presumably functionally optimal composition of isochores” [27]. An alternative proposal for the formation and maintenance of isochores, which states that “biased gene conversion (BGC) is probably the most likely cause of isochores” [7], is probably more reasonable but requires further confirmation. However, the existence and the importance of BGC are not disputed.

In this study, the gene density pattern of LHGRs in the pig genome is found to be identical to the pattern of isochores found in many other species [2]; i.e., there is a regular increase from GC-poor to GC-rich LHGRs (Figure 5). Despite of a much higher gene density in GC-rich than in GC-poor LHGRs, a relatively low gene density is found in the GC-richest LHGRs (see GC content from 55% to 64% in Figure 5). In addition, two peaks of gene density are present: i.e., one peak resides in the GC-content of 50%–51% and the other in 54%–55%. A classical explanation for the high gene density in the GC-rich region is a direct consequence of BGC [28]–[30]. GC-bias in the mismatch repair machinery often leads to gene conversion bias favoring GC-alleles to AT-alleles and, thus, a high level of recombination should be GC-rich [31]–[33]. In addition, when gene transcription promotes DNA recombination [34]–[36], gene regions should be more subject to BGC and thus have a higher GC content. However, the highest GC content region does not have the highest gene density: What factors then lead to this contradiction? One possible explanation is that the time and energy consumption of gene transcription is too high for the organismal body when the gene region has an exceedingly high GC content [37], [38]. Hence, according to the time- and energy-saving organization of the genome, a high GC-content region often does not represent a high gene density. Therefore, based on the previous two explanations, a high gene density resides in a high GC content region, rather than the highest GC content region. However, this model can only account for one of the two gene density peaks in the GC-rich region (54%–55%), and the other peak of gene density locating in a slightly biased GC-rich region (50%–51%) needs to be further explained. To our knowledge, some authors [39], [40] proposed that GC content is positively correlated with the gene expression level, while others [41]–[43] reached a distinct result: GC content is weakly positively or even negatively correlated with gene expression. The two entirely different conclusions were probably due to the slightly biased GC-rich region (50–51%). Hence, we hypothesize that the GC content and gene density are both correlated with the gene expression levels, and the other peak of gene density is constrained by the gene expression levels in the slightly biased GC-rich region. However, even though this hypothesis may be true, we still know little about the two peaks of gene density in the corresponding GC content regions. We hope that further research on these scenarios would be carried out in the near future to identify the reasons for the generation of the two gene density peaks.

In addition, the small number of pig genes concentrated in the GC-rich LHGRs suggests that GC-rich LHGRs may be more likely to harbor genes. Consequently, LHGRs or isochores could be used for *in silico* gene identification. The same is true for the prediction of repeats. Furthermore, repeat identification could be improved by considering LHGRs instead of moving windows, since repeats depend heavily on the GC content of the LHGRs. In fact, Carpena *et al.* [44] showed that the predictive effect of the coding proportion in a sequence is better when isochores, rather than moving windows, are used. Related gene prediction tools, such as ZCURVE [45] and GS-Finder [46], have been developed and were found to perform well.

## Materials and Methods

### LHGR and isochore assignments

The high-coverage Sscrofa9 assembly for chromosomes 1 to 18 and X of the pig genome was downloaded from the Ensembl database (http://www.ensembl.org/index.html, version 56, released in Sep. 2009), while the human genome was downloaded from UCSC (http://hgdownload.cse.ucsc.edu/goldenPath/hg18/chromosomes/). The genome sizes for the pigs and humans are 2.26 and 3.08 Gb, respectively. A PERL script was written to calculate the GC content of each pig chromosome.

The GC boundaries of each LHGR family were defined according to Bernardi's proposal [10]: two types of GC-poor LHGRs — L1 (<37%) and L2 (37%–41%), and three types of GC-rich LHGRs — H1 (41%–46%), H2 (46%–53%), and H3 (>53%).

A windowless tool, GC-Profile (http://tubic.tju.edu.cn/GC-Profile/) [11], was applied to provide an intuitive survey of the heterogeneity in the pig genome through z' curves [19] based on the Z curve method [47], [48]. At the same time, GC-Profile recursively partitioned the input sequence into two subsequences, left and right, by searching for the position producing the maximum quadratic divergence 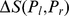 based on the genome order index 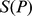. The definitions of the two values are described as follows: 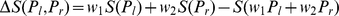, 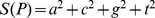, where 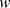 is the weight coefficient, and 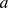, 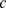, 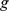, and 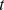 represent the frequencies of the four nucleotide bases A, C, G, and T, respectively. The segmentation procedure was continued until the halting parameter was less than the given threshold 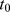, or the resulting sub-sequence was shorter than the given minimum length 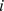. In this work, a total of 24 groups of 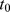 and 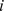 were used in GC-Profile to divide the pig genome. For each group of resulting LHGR families, the average standard deviation (SD) of the GC content was calculated, and both 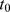 and 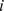 were determined according to the plot variances of the SD values. In addition, to emphasize the overall compositional characteristics of a chromosome, gaps shorter than 1% of the chromosome were ignored, the others were reserved, and then the segmental algorithm was applied to the contigs, which were the original sequences segmented by those unfiltered gaps.

The GC content variance of a LHGR was measured by the homogeneity index 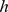 [4], defined by 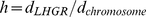, where 
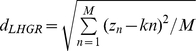
 and 
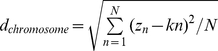
, where 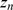 and 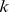 denoted the distribution of base and the slope of the fitted straight line, respectively. If 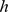 is far less than 1, the GC content of the LHGR could be considered relatively constant compared to that of the whole chromosome. Only when 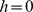 can the GC content of the LHGR be considered absolutely constant. Accordingly, the lower the 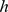 value is, the higher the homogeneity of the LHGR becomes. In this study, the 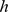 values of isochores were found to be less than 0.2, which is consistent with the study of Zhang *et al.* [4].

### Analysis of Compositional Distribution of Genes

A total of 2,785 pig protein-coding gene annotations and sequences were retrieved from Ensembl 56 using a BioMart tool [49], and the GC3 of each gene was calculated. The genes and LHGRs were then assigned window numbers according to their locations when a 1 Mb non-overlapping window slid along the chromosome. The compositional distributions of the pig coding genes were determined by the two indices: the GC3 of the genes and the GC content of the host LHGRs. Whichever index was chosen, the gene density was defined as gene number per Mb window.

### Identification of repeats in LHGRs

Repeat information in LHGR sequences was detected by the REPEATMASKER mail server (University of Washington Genome Center, Seattle, http://ftp.genome.washington.edu/cgi-bin/RepeatMasker, Repbase 20090604). There were 89 LINEs for the pig species in the Repbase [24]. Ultimately, 120,870 LINEs in the 2,041 LHGRs were used to calculate the LINE density (LINE numbers per Mb window) within different LHGR families. Due to the limited Alu data available for the pigs in Repbase, the Alu density along the LHGRs was ignored in this study.

## Supporting Information

Figure S1 Relationship between the GC content of LHGRs and the gene density in the chromosome 12 of pig. The region between two segmentation points on the z' curve represents one LHGR, and the GC content of this LHGR is illustrated in the corresponding site in the lower figure.
(0.09 MB EPS)

Figure S2 The distribution of GC difference(ΔGC) between neighboring LHGRs is shown for five LHGR families, as well as the total LHGRs. The plot and bar within each box indicate the average and median of ΔGC, respectively, in each family. Among the five families, the ΔGC values for H3 (median 5.78, mean 6.59) are the largest, whereas the ΔGC values for L1 (median 2.47, mean 3.16) are the lowest. The mean of the total ΔGC is 4.24 and the median is 3.58.
(0.04 MB EPS)

Figure S3 GC content of host LHGR vs. GC3 of gene (r^2^ = 0.35, p<10^−6^). A total of 2,785 protein coding genes were included in the comparison. The ellipse shows 95% confidence intervals.
(0.26 MB EPS)

Figure S4 Distribution of gene numbers according to the GC contents of host LHGRs. The fewest genes resided in H3 LHGRs compared with other families.
(0.05 MB EPS)

Table S1 The coordinates, lengths, GC levels, ΔGC, SD, families, types, and h values of the pig LHGRs. ΔGC indicates the difference in GC content between neighboring LHGRs. SD represents for the average standard variance of the GC content in the family to which the LHGR belongs.
(0.44 MB XLS)
